# Supplementary material for: Cytotoxic Effect Induced by Sicilian Oregano Essential Oil in Human Breast Cancer Cells
Source: Cells. 2023 Nov 29;12(23):2733. doi: 10.3390/cells12232733 (PMC10706043; doi:10.3390/cells12232733)
Supplement: Supplementary file 1 [file cells-12-02733-s001.zip › cells-2703156-supplementary.pdf]

Supplemental Materials and Methods

Human bronchial epithelial cell line cell line 16HBE14o was purchased from the American Type Culture Collection. They were grown as monolayers in DMEM medium supplemented with 10 % (v/v) heat inactivated Fetal bovine serum (FBS), antibiotic-antimycotic (100 U/mL penicillin, 100 μg/mL streptomycin and 250 ng/mL amphotericin B),2 mM glutamine and 1 % non-essential amino acids, in a humidified atmosphere containing 5 % CO_2 at 37 °C. Cells were detached from the substrate using 10X trypsin-EDTA solution (5 mg/mL trypsin and 2 mg/mL EDTA) diluted in PBS (Phosphate buffered saline: 137 mM NaCl, 2.68 mM KCl, 10 mM Na_2HPO_4, KH_2PO_4 1.76 mM, pH 7.4) at 0.25 X concentration. Cells were seeded in 6 (2×10^5 cells/2 mL), 24 (6×10^4 cells/1 mL), and 96 well (8×10^3 cells/200 μL). After plating, the cells were placed for 24 h in an incubator at 37 °C and subsequently treated with the various compounds and at various times, as indicated in the results.

Supplemental Results

The effect of OEO on cell viability was evaluated on a normal cell line 16HBE14o, a human bronchial epithelial cell line. Experimental data show that this line is less sensitive to the OEO effect (Fig. S1A and B). Toxicity is especially evident with 0.2 % OEO. Light microscopic observations confirm that 0.05 % OEO induces modest effects in 16HBE14o compared to breast cancer lines examined. Treatment of 16HBE14o with 0.05 % OEO for 24 h did not result in an evident loss of mitochondrial membrane potential (Fig. S1C).

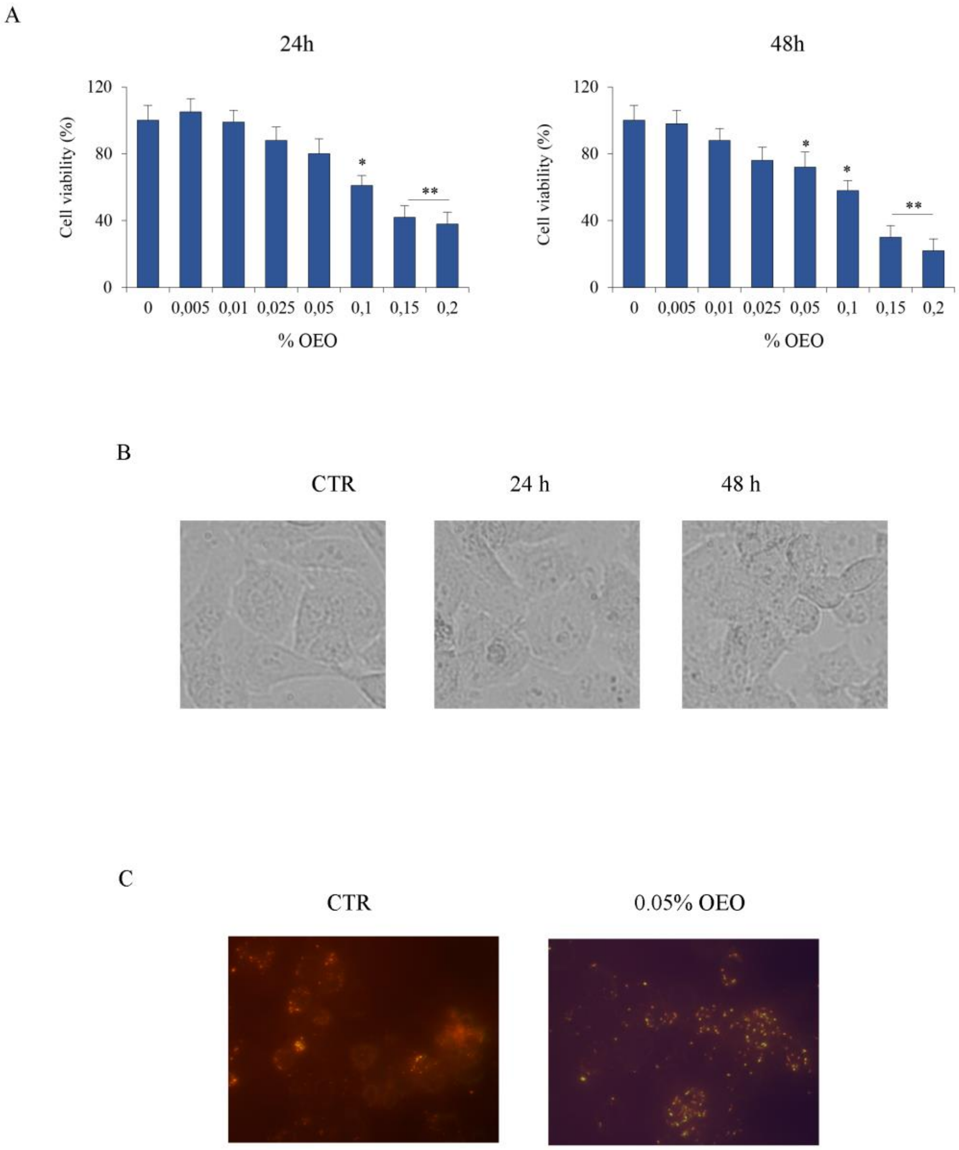

Figure S1. Cytotoxic effect exerted by OEO on 16HBE14o. (A) Effects of OEO on cell viability. Cells were treated with different percentage of OEO for 24h and 48h. Cell viability was assessed by MTT, as described in Methods. (B) Morphologic changes of 16HBE14o cells observed under light microscopy at 400 X magnification. (C). To evaluate the effects of OEO on mitochondrial membrane potential, cells were treated for 24 h with 0.05 % OEO. At the end cells were incubated with JC-1 fluorochrome and fluorescent cells were visualized with a OPTIKA microscope at 400 X magnification, as indicated in Methods. Results are representative of three independent experiments. In (A) values are the means of three independent experiments ±S.D. *$p < 0.05$, **$p < 0.01$ versus untreated control.
